# Supplementary material for: Exceptionally low genomic diversity in the underutilised legume Kersting’s groundnut
Source: Nat Commun. 2025 Jun 4;16:5183. doi: 10.1038/s41467-025-60494-x (PMC12137951; doi:10.1038/s41467-025-60494-x)
Supplement: Supplementary file 2 — Description of Additional Supplementary Files [file 41467_2025_60494_MOESM2_ESM.pdf]

Description of Additional Supplementary Files

File Name: Supplementary Data 1
Description: Output from misa identifying microsatellites in the genome sequence.

File Name: Supplementary Data 2
Description: Sample data and resequencing statistics for Kersting's groundnut.

File Name: Supplementary Data 3
Description: Sample, sequencing, and mapping data for cowpea and lablab.
