# Supplementary material for: Exceptionally low genomic diversity in the underutilised legume Kersting’s groundnut
Source: Nat Commun. 2025 Jun 4;16:5183. doi: 10.1038/s41467-025-60494-x (PMC12137951; doi:10.1038/s41467-025-60494-x)
Supplement: Supplementary file 6 — Reporting Summary [file 41467_2025_60494_MOESM6_ESM.pdf]

nature portfolio

Corresponding author(s): MARK CHAPMAN

Last updated by author(s): Nov 15, 2024

Reporting Summary

Nature Portfolio wishes to improve the reproducibility of the work that we publish. This form provides structure for consistency and transparency in reporting. For further information on Nature Portfolio policies, see our Editorial Policies and the Editorial Policy Checklist.

Statistics

For all statistical analyses, confirm that the following items are present in the figure legend, table legend, main text, or Methods section.

n/a	Confirmed	
☐	☒	The exact sample size (*n*) for each experimental group/condition, given as a discrete number and unit of measurement
☒	☐	A statement on whether measurements were taken from distinct samples or whether the same sample was measured repeatedly
☐	☒	The statistical test(s) used AND whether they are one- or two-sided<br>*Only common tests should be described solely by name; describe more complex techniques in the Methods section.*
☒	☐	A description of all covariates tested
☐	☒	A description of any assumptions or corrections, such as tests of normality and adjustment for multiple comparisons
☒	☐	A full description of the statistical parameters including central tendency (e.g. means) or other basic estimates (e.g. regression coefficient) AND variation (e.g. standard deviation) or associated estimates of uncertainty (e.g. confidence intervals)
☐	☒	For null hypothesis testing, the test statistic (e.g. *F*, *t*, *r*) with confidence intervals, effect sizes, degrees of freedom and *P* value noted<br>*Give P values as exact values whenever suitable.*
☒	☐	For Bayesian analysis, information on the choice of priors and Markov chain Monte Carlo settings
☒	☐	For hierarchical and complex designs, identification of the appropriate level for tests and full reporting of outcomes
☒	☐	Estimates of effect sizes (e.g. Cohen's *d*, Pearson's *r*), indicating how they were calculated

Our web collection on statistics for biologists contains articles on many of the points above.

Software and code

Policy information about availability of computer code

Data collection	NO SPECIFIC SOFTWARE WAS USED FOR DATA COLLECTION

Data analysis	Genome assembly, annotation and analysis: De novo genome assembly of the PacBio HiFi reads was performed using Hifiasm (v0.19.9-r616); Omni-C and short RNA reads were trimmed using Trim galore (v0.6.7); cleaned Omni-C reads were pre-processed with Juicer (v1.5.7), followed by scaffolding through 3D-DNA pipeline (v180922); completeness of the genome assembly was evaluated using BUSCO (v5.7.1); Repetitive elements, including transposable elements, simple repeats and small RNA were identified using RepeatModeler (v2.0) and soft-masked using RepeatMasker (v 4.1.0); RNA short reads were aligned to the genome with HISAT2 (v2.2.1); raw Nanopore sequencing signals were pre-processed using Guppy (v6.4.6)and aligned to the genome using Minimap2 (v2.24-r1122); RNAseq alignment files were merged using SAMtools (v1.17), subjected to de novo transcriptome assembly using Trinity (v2.1.1) and a preliminary gene annotation file generated using PASA (v2.4.1); Funannotate (v1.8.9) was used to perform ab initio gene prediction; transfer RNAs (tRNA) were identified using tRNAscan-SE (v.2.0.11); long non-coding RNAs were identified using CPC2 (v1.0.1) and CNCI (v2); functional annotation was performed using InterProScan (v5.63-95.0); synteny analysis was performed using MCScan (v0.9.12); gene families were identified using Orthofinder (v2.3.3); gene family expansion and contraction was performed using CAFE5 (v5.1.0); Gene Ontology enrichment was carried out using GSEApy (v 1.1.0). The LTR assembly index (LAI) was assessed by LTR_retriever (v3.0.1); LTR retrotransposons were identified using LTR_FINDER (v1.2) and LTR_retriever (v3.0.1). Microsatellites were identified using misa ver 1.0 (https://github.com/cfljam/SSR_marker_design/blob/master/misa.pl),<br><br>Resequencing, clustering and polymorphism analysis: Illumina sequencing reads were trimmed using Trimmomatic (v 0.36) and mapped to the reference genome using bowtie2 bowtie2 (v 2.3.1); samtools (v 1.20) was used to convert between sam and bam filetypes; bcftools (within samtools) was used to generate VCF files, call and filter data in the VCFs; PLINK (v 1.9) was used to identify SNPs in LD; vcftools (v 0.1.16) was used to remove SNPs in LD and to calculate genetic diversity (π) and population divergence (Fst); VCF2Dis (https://github.com/BGI-shenzhen/VCF2Dis; v 1.50) was used to generate distance matrices; STRUCTURE ver. 2.3.4 was used to estimate sample clustering.

For manuscripts utilizing custom algorithms or software that are central to the research but not yet described in published literature, software must be made available to editors and reviewers. We strongly encourage code deposition in a community repository (e.g. GitHub). See the Nature Portfolio guidelines for submitting code & software for further information.

Data

Policy information about availability of data

All manuscripts must include a data availability statement. This statement should provide the following information, where applicable:

- Accession codes, unique identifiers, or web links for publicly available datasets
- A description of any restrictions on data availability
- For clinical datasets or third party data, please ensure that the statement adheres to our policy

Provide your data availability statement here.

Research involving human participants, their data, or biological material

Policy information about studies with human participants or human data. See also policy information about sex, gender (identity/presentation), and sexual orientation and race, ethnicity and racism.

Reporting on sex and gender	No human participants or data were included in this study
Reporting on race, ethnicity, or other socially relevant groupings	No human participants or data were included in this study
Population characteristics	No human participants or data were included in this study
Recruitment	No human participants or data were included in this study
Ethics oversight	No human participants or data were included in this study

Note that full information on the approval of the study protocol must also be provided in the manuscript.

Field-specific reporting

Please select the one below that is the best fit for your research. If you are not sure, read the appropriate sections before making your selection.

☐ Life sciences ☐ Behavioural & social sciences ☒ Ecological, evolutionary & environmental sciences

For a reference copy of the document with all sections, see nature.com/documents/nr-reporting-summary-flat.pdf

Ecological, evolutionary & environmental sciences study design

All studies must disclose on these points even when the disclosure is negative.

Study description	A reference genome for Kersting's groundnut, an underutilised legume crop, was generated. This was annotated and compared to demonstrate the degree of synteny with two related species. Further samples (25) were sequenced as well as an outgroup, and standard population genomic and phylogenetic methods were applied to identify population clusters and relationships. Genomic regions potentially involved in the differences in seed colour were identified by comparing groups of accessions with different seed colours. To compare genetic diversity in Kerstings Groundnut to other species, publicly available (NCBI SRA) data were used (accessions indicated in the manuscript) as well as from our own labs (also uploaded to SRA).
Research sample	Accession TKg36 from Nigeria was used for reference genome sequencing and annotation. Other samples are listed clearly in the manuscript.
Sampling strategy	Plants were grown in greenhouses and DNA/RNA extracted from leaves.
Data collection	Sequencing data were generated from samples using standard approaches at a service provider (Novogene)
Timing and spatial scale	DNA and RNA samples were taken from TKg36 in May to October 2023 from a plant grown in the CUHK greenhouse. Resequencing samples were grown at the University of Southampton in autumn 2022 to summer 2023. Samples from Benin, Burkina Faso and Ghana were grown in the field in autumn 2022.
Data exclusions	No data was excluded, except when quality control removed poor sequencing reads (as indicated in the manuscript)
Reproducibility	Reproducibility is not relevant
Randomization	Randomisation is not relevant
Blinding	Blinding is not relevant

Did the study involve field work? ☐ Yes ☒ No

Reporting for specific materials, systems and methods

We require information from authors about some types of materials, experimental systems and methods used in many studies. Here, indicate whether each material, system or method listed is relevant to your study. If you are not sure if a list item applies to your research, read the appropriate section before selecting a response.

Materials & experimental systems

n/a	Involved in the study	
☒	☐	Antibodies
☒	☐	Eukaryotic cell lines
☒	☐	Palaeontology and archaeology
☒	☐	Animals and other organisms
☒	☐	Clinical data
☒	☐	Dual use research of concern
☐	☒	Plants

Methods

n/a	Involved in the study	
☒	☐	ChIP-seq
☒	☐	Flow cytometry
☒	☐	MRI-based neuroimaging

Dual use research of concern

Policy information about dual use research of concern

Hazards

Could the accidental, deliberate or reckless misuse of agents or technologies generated in the work, or the application of information presented in the manuscript, pose a threat to:

No	Yes	
☒	☐	Public health
☒	☐	National security
☒	☐	Crops and/or livestock
☒	☐	Ecosystems
☒	☐	Any other significant area

Experiments of concern

Does the work involve any of these experiments of concern:

No	Yes	
☒	☐	Demonstrate how to render a vaccine ineffective
☒	☐	Confer resistance to therapeutically useful antibiotics or antiviral agents
☒	☐	Enhance the virulence of a pathogen or render a nonpathogen virulent
☒	☐	Increase transmissibility of a pathogen
☒	☐	Alter the host range of a pathogen
☒	☐	Enable evasion of diagnostic/detection modalities
☒	☐	Enable the weaponization of a biological agent or toxin
☒	☐	Any other potentially harmful combination of experiments and agents

Plants

Seed stocks	Seed were obtained from IITA (International Institute of Tropical Agriculture) or are housed at the University of Development Studies (UDS), Institut de l'Environnement et de Recherches Agricoles (INERA)or Laboratory of Applied Ecology, University of Abomey-Calavi.
Novel plant genotypes	No novel genotypes were included in this work
Authentication	No authentication was used except for assessing morphologically whether the species was as expected.
